# Clinical Practice Guidance: Surveillance for phaeochromocytoma and paraganglioma in paediatric succinate dehydrogenase gene mutation carriers

**DOI:** 10.1111/cen.13926

**Published:** 2019-01-29

**Authors:** Mei Yin Wong, Katrina A. Andrews, Benjamin G. Challis, Soo‐Mi Park, Carlo L. Acerini, Eamonn R. Maher, Ruth T. Casey

**Affiliations:** ^1^ Department of Diabetes and Endocrinology Cambridge University Hospital NHS Foundation Trust Cambridge UK; ^2^ East Anglian Medical Genetics Service Cambridge University Hospitals NHS Foundation Trust Cambridge UK; ^3^ Department of Paediatrics University of Cambridge Cambridge UK; ^4^ Department of Medical Genetics University of Cambridge Cambridge UK; ^5^ NIHR Cambridge Biomedical Research Centre and Cancer Research UK Cambridge Centre Cambridge UK

## Abstract

The succinate dehydrogenase (SDH) enzyme complex functions as a key enzyme coupling the oxidation of succinate to fumarate in the citric acid cycle. Inactivation of this enzyme complex results in the cellular accumulation of the oncometabolite succinate, which is postulated to be a key driver in tumorigenesis. Succinate accumulation inhibits 2‐oxoglutarate‐dependent dioxygenases, including DNA and histone demethylase enzymes and hypoxic gene response regulators. Biallelic inactivation (typically resulting from one inherited and one somatic event) at one of the four genes encoding the SDH complex (*SDHA/B/C/D*) is the most common cause for SDH deficient (dSDH) tumours. Germline mutations in the *SDHx* genes predispose to a spectrum of tumours including phaeochromocytoma and paraganglioma (PPGL), wild type gastrointestinal stromal tumours (wtGIST) and, less commonly, renal cell carcinoma and pituitary tumours. Furthermore, mutations in the *SDHx* genes, particularly *SDHB*, predispose to a higher risk of malignant PPGL, which is associated with a 5‐year mortality of 50%. There is general agreement that biochemical and imaging surveillance should be offered to asymptomatic carriers of *SDHx* gene mutations in the expectation that this will reduce the morbidity and mortality associated with dSDH tumours. However, there is no consensus on when and how surveillance should be performed in children and young adults. Here, we address the question: “What age should clinical, biochemical and radiological surveillance for PPGL be initiated in paediatric *SDHx* mutation carriers?”.

## 1 INTRODUCTION

Prior to the turn of this century, it was commonly considered that 10% of phaeochromocytomas and paragangliomas (PPGLs) were familial. However the recent molecular revolution has brought with it an understanding that PPGL have a rich hereditary background, as 30% of PPGL are now known to be familial.^1^ Germline mutations in the *SDHx *genes account for 30‐40% of hereditary PPGL cases and mutations in the *SDHB* gene in particular predict a higher risk of malignant potential.^2^ Although there is is general agreement that biochemical and imaging surveillance should be offered to asymptomatic carriers of *SDHx* gene mutations in the expectation that this will reduce the morbidity and mortality associated with SDH deficient tumours,^3^ there is at present no consensus on when and how surveillance should be performed in children and young adults. Here, we address the question: "What age should clinical, biochemical and radiological surveillance for PPGL be initiated in paediatric *SDHx* mutation carriers?".

## 2 METHODS

In order to address this important clinical question, a thorough review of the literature was performed. MEDLINE was searched via PubMed using the following search terms; (a) SDH or succinate dehydrogenase and (b) child or children or paediatric or adolescent and (c) tumour or cancer or paraganglioma or phaeochromocytoma or PPGL or GIST. This search yielded 413 results, of which 43 papers were relevant. An additional five papers were identified through the initial 43 manuscripts. Papers were included in this review if they contained paediatric index cases, detailed phenotypes including age of presentation, and in the case of having both index and nonindex or asymptomatic carriers, and a clear discrimination between the two was required for inclusion in this literature review. A second search was performed to identify the literature addressing the issue of surveillance in paediatric *SDHx* carriers and the term “surveillance” was added to the above search terms. This search yielded 275 results, of which 11 were relevant.

### 2.1 What is the prevalence of related disease in childhood *SDHx* carriers?

**Figure 1:**
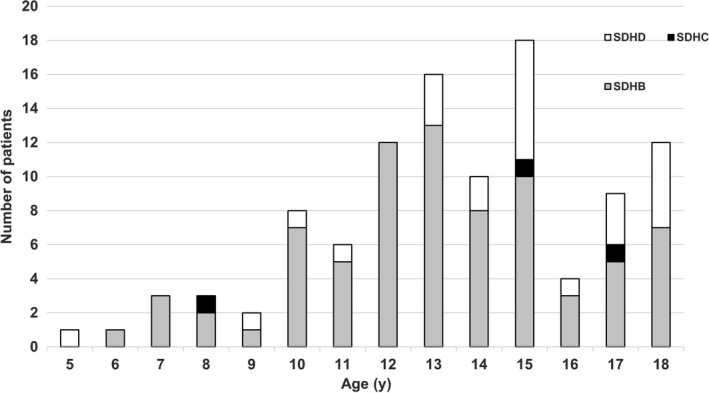
Illustrates the individual succinate dehydrogenase gene subunit variant carried by the index cases and the age of presentation with a phaeochromocytoma and paraganglioma (PPGL)

Whilst there are several reports of tumour development in paediatric *SDHx* mutation carriers, the prevalence of disease among paediatric nonprobands remains low. A review of the literature identified 105 paediatric (<18 years of age) index cases for which detailed data on the phenotype were available. A review of the genotype information demonstrated that 77 (73.3%) patients had a germline pathogenic *SDHB *variant, 25 (23.8%) cases a pathogenic *SDHD *variant and three cases (2.9%) a pathogenic *SDHC* variant (Figure 1). The variant type was available for 93 patients and included 20 (21.6%) nonsense variants, 28 (30.1%) missense variants, 13 (13.9%) splice site variants, 26 (27.9%) frameshift variants. A copy number variant was identified in 6 (6.4%) cases (one gene duplication, five exonic deletions and one whole gene deletion). Notably, the frequency of reported truncating variants (nonsense/frameshift and splice site variants) identified in this paediatric population was 72% compared to a reported frequency of 52% in an adult population presenting with PPGL.^1^

The mean age of this cohort was 13.5 years (range 5‐18 years). There was no significant difference in the mean age at presentation with PPGL in those patients with germline *SDHB* variants (13.1 years) compared to those with *SDHC* (13.3 years) or *SDHD* variants (14.6 years) (*P* = 0.78), and there was no significant difference in the mean age at presentation of those patients presenting with malignant tumours vs those presenting with localized tumours (12.8 vs 13.4 years, *P* = 0.9). Over a third of identified cases (34.3%) had multiple synchronous or metachronous PPGL (Table 1 and Table S1 in supplementary data). An extra‐adrenal paraganglioma was reported in 65 (61.9%), 31 (29.5%) patients presented with a phaeochromocytoma, 8 (7.6%) patients presented with both tumour types, and for one case the exact tumour type was not stated. The secretory status of the tumour was reported for 73 patients: 63 (86.3%) patients with biochemically functioning tumours and 10 (13.7%) cases had a nonsecretory tumour. Four patients in this cohort also developed a non‐PPGL tumour including one renal cell carcinoma, one renal oncocytoma, one nephrogenic adenoma and one pituitary tumour (Table 1 and Table S1). Malignant tumours were reported in 26 patients (24.8%), and the majority of malignant cases were identified in patients with a pathogenic *SDHB* germline variant (22 patients, 84.6%). Of note, this is a lower figure than what King et al^4^ found in their study of metastatic PPGL related to primary tumour development in childhood and adolescence, where 85.2% (n = 23) of their paediatric and adolescent patients with *SDHB* mutations developed metastatic disease. This review of the literature highlights not only the importance of identifying a germline mutation in children presenting with PPGL, but also the possibility of *SDHx* mutation penetrance at a young age. However, an appropriate surveillance protocol must balance the occurrence of paediatric tumour development against the increasing evidence that the penetrance of *SDHB* is lower than originally reported and the potential adverse effects of screening.

**Table 1:** Summary of paediatric index cases with a succinate dehydrogenase gene subunit (*SDHx*) mutation reported in literature (data based on references^5^, ^6^, ^7^, ^8^, ^9^, ^10^, ^11^, ^12^, ^13^, ^14^, ^15^, ^16^, ^17^, ^18^, ^19^, ^20^, ^21^, ^22^, ^23^, ^24^, ^25^, ^26^, ^27^, ^28^, ^29^, ^30^, ^31^, ^32^, ^33^, ^34^, ^35^, ^36^, ^37^, ^38^, ^39^, ^40^, ^41^, ^42^, ^43^, ^44^, ^45^, ^46^, ^47^, ^48^, ^49^, ^50^, ^51^, ^52^)

*SDHx* subunit gene	
*SDHB*	77 (73.3%)
*SDHC*	3 (2.9%)
*SDHD*	25 (23.8%)
Variant type	
NA	12
Nonsense	20 (21.6%)
Missense	28 (30.1%)
Splice site	13 (13.9%)
Frameshift	26 (27.9%)
Copy number variation	6 (6.4%)
Tumour type	
PGL	65 (61.9%)
PCC	29 (27.6%)
Both	10 (9.5%)
Not known	1 (1.0%)
Non‐PPGL	4
Multifocal disease	
Present	36 (34.3%)
Absent	69 (65.7%)
Functional status	
NA	32
Functioning	63 (86.3%)
Nonfunctioning	10 (13.7%)
Malignant disease	
Present	26 (24.8%)
Absent	79 (75.2%)

NA, not available; PCC, phaeochromocytoma; PGL, paraganglioma; PPGL, phaeochromocytoma and paraganglioma.

### 2.2 What are the current recommendations for the age at which to commence surveillance for *SDHx *mutation carriers?

The Endocrine Society recommends that surveillance should comprise annual biochemistry (urinary or plasma metanephrines) and sporadic cross‐sectional imaging of the skull base, neck, thorax, abdomen and pelvis (MRI is the preferred radiation sparing imaging modality).^3^ There are no recommendations as to the lower age limit of genetic testing of children in *SDHx* mutation families. In the UK, genetic testing for inherited neoplasia syndromes is usually conducted around the time when clinical, biochemical and radiological surveillance would begin.^53^ It is also important to note that as *SDHD* variants have a preferential paternal transmission pattern of inheritance, clinical surveillance is only recommended for those carriers that inherit an *SDHD* variant from their father.

To our knowledge, no guidance exists to inform the most appropriate starting age for clinical, biochemical and radiological surveillance in paediatric *SDHx* mutation carriers. A review of the literature identified 13 manuscripts that attempted to address this issue. Eight studies argued for surveillance to start before or at 10 years including one expert opinion,^54^ three case series with less than five patients,^6^, ^55^, ^56^ three retrospective studies with 32, 92 and 116 patients, respectively^5^, ^57^, ^58^), and one case‐control study with 241 patients.^59^ Three reports recommended starting from the second decade of life (one systematic review of 95 papers suggesting a start age between 11‐20 years, one retrospective study with 91 patients suggesting an age of 27.1 years based on HNPGL penetrance calculations and one case series of three families suggested a starting age of 18 years for HNPGL screening).^15^, ^60^, ^61^ However, two further studies suggested that the starting age should either be between 5 and 10 years or alternatively a minimum of one decade before the earliest age of disease onset in that kindred (two expert opinions).^62^, ^63^

When considering at what age a screening programme should be commenced it is relevant to consider (a) what ages have *SDHx*‐related tumours occurred? (b) what is the estimated risk at different ages? and (c) what is the risk threshold at which screening is deemed appropriate? Ideally, this decision would be based on a robust cost‐benefit analysis (including the health costs of false positive screening diagnoses) but for rare disorders such *SDHx*‐related tumours evidence is necessarily limited and therefore decisions will involve a major element of expert judgement.

Recent studies, focused on more accurately predicting the clinical penetrance of *SDHx* genes, have adopted methods to control for ascertainment bias and suggest an estimated clinical penetrance of around 20% by age 50 years for *SDHB *mutation carriers.^64^

In the largest study of *SDHB* and *SDHD *mutation carriers yet reported, Andrews et al^65^ estimated the risks of PPGL and head and neck paraganglioma (HNPGL) by ages at 5, 10, 16 and 18 years in *SDHB* mutation carriers (n = 598 with clinical information available) and in *SDHD* carriers (n = 137 with clinical information available) (Table 2). There are some limitations to this analysis. Firstly, the figures include probands who are affected with the disease and therefore will increase penetrance estimates, and therefore, the analysis was also performed with probands excluded (Table 2). The second limitation of this study is that as these are clinical penetrance risks, they will not include asymptomatic tumours that might have been detected by screening. Using a different methodology, Benn et al recently estimated the lifetime penetrance of pathogenic *SDHA, SDHB *and *SDHC* variants by adopting an algorithm which compared allelic frequencies in 1815 cases (including cases from ref. [65]) vs controls from ExAC (http://exac.broadinstitute.org). The estimated lifetime penetrance of pathogenic *SDHA* and *SDHC* variants was low (1.7% and 8.3%, respectively), whilst the penetrance for *SDHB* was similar to that previously reported at 20.2%.^66^ The authors also speculated that *SDHx* gene penetrance may be directly proportional to the risk of multifocal tumours as *SDHB* gene mutations are more commonly associated with multifocal tumours compared to *SDHA* gene mutations.

**Table 2:** Estimated risk of PPGL and HNPGL for *SDHB* and *SDHD *carriers by age 5, 10, 16 and 18 y, respectively, in data from Andrews et al^65^

*SDHx* subunit gene	Penetrance at age 5 y		Penetrance at age 10 y		Penetrance at age 16 y		Penetrance at age 18 y	
	PPGL	HNPGL	PPGL	HNPGL	PPGL	HNPGL	PPGL	HNPGL
*SDHB* n = 598	0.17% [95% CI 0.0‐0.51]	0%	1.7% [95% CI 0.67‐2.8]	0%	7.6% [95% CI 5.4‐9.8]	0.38% [95% CI 0.0‐0.90]	10.2% [95% CI 7.6‐12.7	0.58% [95% CI 0.0‐1.2]
*SDHD* n = 137	0%	0%	0.28% [95% CI 0.0‐0.82]	0%	3.1% [95% CI 0.062‐6.0]	1.6% [95% CI 0.0‐3.7]	7.0% [95% CI 2.5‐11.3]	3.2% [95% CI 0.063‐6.1]
*SDHB* a n = 371	0%	0%	0.28% [95% CI 0.0‐0.82]	0%	1.2% [95% CI 0.023‐2.4]	0%	2.2% [95% CI 0.56‐3.7]	0.32% [95% CI 0.0‐0.94]
*SDHD* a n = 67	0%	0%	0%	0%	0%	0%	6.4% [95% CI 0.13‐12.3]	1.7% [95% CI 0.0‐4.9]

NB. No confidence intervals are given before the first noncensored event (if no children in the cohort have experienced tumours by the age specified).

HNPGL, head and neck paraganglioma; PPGL, phaeochromocytoma and paraganglioma.

a Nonproband gene carriers only (probands excluded from analysis).

### 2.3 What are the controversies and/or risks of early surveillance?

Several factors complicate the decision when surveillance should be commenced in paediatric *SDHx *mutation carriers. Important considerations include the growing awareness that the lifetime penetrance of *SDHB* mutations is significantly lower (see above) than that estimated when the gene was first identified (originally estimated at 70%‐80%). In addition, surveillance programmes can be disruptive to patients’ lives, requiring them (and their parents) to take time off school or work and incur travel costs (and screening costs if not covered by a national health system or health insurance). The potential for the identification of incidental lesions on imaging raises several ethical and medicolegal questions also. Onwubiko and Mooney^67^ found that 35.7% (n = 86) of paediatric patients attending their level 1 paediatric trauma centre had at least one incidental finding on computed tomography studies of the thorax, abdomen and pelvis. However, only 30 (63.8%) of the clinically significant findings were reported, with follow‐up recommended by the radiologist in 14 cases (29.8%), and only four patients (4.6%) required further investigations. Incidental findings may be associated with a psychological burden and additional health costs.^68^ Finally, there is extremely limited information on the specificity and sensitivity of surveillance modalities for child and adolescent *SDHx* mutation carriers. Though the use of MRI instead of CT imaging reduces exposure to ionizing radiation, there is a lack of conclusive data on the potential long‐term consequences of exposure to the strong magnetic fields employed by MRI.^69^ Some groups have adapted their surveillance programmes at younger ages. Rijken et al^64^ started surveillance in a 7‐year‐old *SDHB *mutation carrier by urinary catecholamines measurements, allowing for surveillance to commence without the stress of imaging or phlebotomy. However, 24‐hour urinary collections can be difficult to obtain in young children. Tufton et al^57^ replaced MRI imaging with ultrasound in children under 10 years of age if they were unable to tolerate MRI. In doing so, the patients could experience the potential benefits of earlier surveillance whilst minimizing the anxiety involved during surveillance. However it must also be considered that ultrasound has a lower sensitivity and specificity compared with MRI in PPGL detection.^3^

The Endocrine Society clearly state that the morbidity associated with *SDHB* gene mutations requires particular attention,^3^ and therefore, more stringent screening efforts may be necessary in order to reduce the morbidity and mortality associated with *SDHB*‐mutated PPGL. It is notable that in the paediatric population studied in this review (similar to adult populations), *SDHB *is the most common *SDHx *subunit gene to be implicated in index cases presenting with PPGL (77/105, 73.3%) and malignant PPGL (22/26, 84.6%).

Finally, parental anxiety may also influence the decision as to when genetic testing should be performed and when clinical surveillance should be commenced and this can be a complex issue to manage in clinical practice. It is clear that when possible, a balance should be struck between being flexible and open to the needs and concerns of a family, whilst still keeping the interests and the long‐term welfare of the child at the forefront.^53^

## 3 CONCLUSION

Despite the occurrence of *SDHx* driven tumours in children, the majority of these tumours present in adulthood. The absolute risks of a “clinical PPGL” in the paediatric age group are estimated to exceed 1% and 5% at ages 10 years and 16 years in *SDHB *mutation carriers and ages 16 and 18 years in *SDHD *carriers, respectively.^65^ Reviewing those paediatric index cases with *SDHx *variants reported in the literature, 9.5% (10/105) of cases presented before the age of 10 years and 70% of those cases were *SDHB* gene mutation carriers. Furthermore, in cases for which detailed phenotypic data on paediatric index cases were available (Table 1 and Supporting Information Table S1), 86.3% (63/73) of tumours reported were secretory and therefore could be diagnosed on biochemical screening.

Importantly, this data would suggest that the approach to clinical surveillance should be tailored to the *SDHx* subunit gene affected, as recent studies would suggest a higher risk of PPGL (and possibly of synchronous and multifocal tumours) with *SDHB* gene mutations. Based on the available data reviewed herein both for asymptomatic *SDHx* carriers and index cases, we propose that for *SDHB* mutation carriers annual physical examination (with blood pressure) and biochemical screening should generally commence at the age of 5 years and at age 10 years for *SDHA*, *SDHC *and *SDHD *carriers. Radiological surveillance using MRI (or ultrasound for those who find MRI intolerable) and MRI of head and neck should be commenced from the age of 10 years for *SDHB *and 15 years for *SDHA*, *SDHC* and *SDHD* carriers and repeated every 2‐3 years if annual biochemical testing is normal. A MRI scan would be performed earlier if biochemical investigations are abnormal (the majority of early onset PPGL are biochemically active). Genetic testing can be offered to families from the ages at which surveillance would be initiated (5 or 10 years). Clinical and genetic investigations should be performed earlier if clinical symptoms develop, and an earlier age for starting MRI surveillance could be adopted in those rare families in which a PPGL has occurred in a child (or young adult) or based on the judgement of an experienced endocrinologist or paediatric endocrinologist. Finally, this clinical review has highlighted the need for a large prospective multicentre study to better inform existing surveillance and management strategies for paediatric and adolescent *SDHx* mutation carriers.

## CONFLICT OF INTEREST

The authors have no conflict of interests and nothing to declare.
