# Supplementary material for: Clinical Practice Guidance: Surveillance for phaeochromocytoma and paraganglioma in paediatric succinate dehydrogenase gene mutation carriers
Source: Clin Endocrinol (Oxf). 2019 Jan 29;90(4):499–505. doi: 10.1111/cen.13926 (PMC6850004; doi:10.1111/cen.13926)
Supplement: Supplementary file 1 [file CEN-90-499-s001.docx]

**Supplementary data**

**Table S1: A detailed review of paediatric index cases with succinate dehydrogenase gene subunit (*SDHx*) mutations and their associated phenotypes reported in the literature**

*NA = Not available*

*PGL = Paraganglioma*

*PCC = Phaeochromocytoma*

*HNPGL = Head and neck paraganglioma*

| **Authors** | **Age of proband at first onset** | **Genetic mutation** | **Mutation type (protein change)** | **Symptoms** | **Phenotype** |
| --- | --- | --- | --- | --- | --- |
| **SDHB Mutations** | | | | | |
| Srirangalingam et al (2008)(4) | 10 years | c.141G>A | (p.Trp47X) | Not known | Non-functioning abdominal PGL, followed by papillary renal cell carcinoma (type II) 16.5 years later and subsequently metastatic papillary renal cell carcinoma (type II) a year after. |
|  | 18 years | c.136C>T | (p.Arg46X) | Not known | Functioning abdominal PGL |
|  | 14 years | c.72+1G>T | NA | Not known | Functioning PCC |
|  | 11 years | c.137G>A | (p.Arg46Gln) | Not known | Functioning abdominal PGL, PCC and thoracic PGL, with metastases |
| Prodanov et al (2009)(5) | 8 years | c.418G>T | (p.Val140Phe) | Three year history of paroxysmal sweating, headache, nausea, vomiting, fatigue and weight loss | Malignant functioning PGL |
| Imamura et al (2016)(6) | 6 years | c.423+1G>A | NA | Convulsions following a severe headache, on a background of a one month history of headache and paroxysmal sweating | Functioning PGL |
| Benn et al (2003)(7) | 7 years | c.136C>T | (p.Arg46X) | Not known | Functioning PCC, subsequent second primary |
|  | 12 years | IVS4-1G>A | NA | Not known | Lung metastases with abdominal mass consistent with PCC/PGL |
|  | 15 years | c.88delC | (p.Gln30Argfs) | Not known | Functioning thoracic PGL |
| Neumann et al (2004)(8) | 13 years | c.213C>T | (p.Arg27X) | Not known | Functioning extra-adrenal PCC |
|  | 14 years | c.270C>G | (p.Arg46Gly) | Not known | Functioning extra-adrenal PCC |
|  | 15 years | c.270C>G | (p.Arg46Gly) | Not known | Functioning extra-adrenal PCC |
|  | 15 years | c.328T>C | (p.Leu65Pro) | Not known | Functioning adrenal and extra-adrenal PCC |
|  | 17 years | c.394T>C | (p.Leu87Ser) | Not known | Functioning PCC |
|  | 10 years | c.436G>A | (p.Cys101Tyr) | Not known | Functioning extra-adrenal PCC |
|  | 16 years | c.847delTCTC | NA | Not known | Functioning extra-adrenal PCC |
|  | 12 years | c.881C>A | (p.Cys249X) | Not known | Functioning PCC |
| Bockenhauer et al (2008)(9) | 7 years | c.158G>A | (p.Gly53Glu) | Two month history of diaphoresis and headaches (associated with vomiting and eye pain) | Functioning PCC |
| Astuti et al (2001)(10) | 10 years | c.402C>T | (p.Arg91X) | Not known | Functioning extra-adrenal PCC |
| Astuti et al (2003)(11) | 11 years | c.660-661insT | (p.Asp221X) | Not known | Functioning PCC |
|  | 13 years | c.380T>A | (p.Ile127Asn) | Not known | Functioning PCC |
| Graham et al (2014)(12) | 12 years | c.79C>T | (p.Arg27X) | Six month history of worsening headaches (occasionally accompanied by light-headedness, nausea, palpitations and flushing) and weight loss | Functioning PCC |
| Santiago et al (2010)(13) | 13 years | c.418G>T | (p.Val140Phe) | Intermittent headaches, palpitation, vomiting, flank pain and poor weight gain | Functioning peri-aortic PGL |
| Luiz et al (2013)(15) | 12 years | c.725G>A | (p.Arg242His) | Haemorrhagic stroke with sudden onset headache, sweating, hemiparesis and reduced consciousness on a four year background of frequent headaches | Two abdominal functioning PGLs, preceding metastatic disease years later |
| McDonnell et al (2004)(33) | 12 years | c.118A>G | (p.Lys40Glu) | Hypertensive crisis and convulsions on anaesthetic induction, with a two year history of headaches, anxiety, palpitations with exercise, diaphoresis | Functioning PCC |
| Bayley et al (2009)(34) | 12 years | c.201-4429_287-933del | (p.Cys68HisfsX21) | Not known | Malignant extra-adrenal PGL with bone metastases, and a pituitary tumour |
|  | 18 years | c.201-4429_287-933del | (p.Cys68HisfsX21) | Not known | Juxtaglomerular PGL |
| Park et al (2017)(35) | 15 years | c.603G>A | (p.Trp201X) | Right flank pain, headache, diaphoresis, palpitations, weight loss | Functioning bilateral PCC |
| Pandit et al (2016)(36) | 13 years | c.136C>T | (p.Arg46X) | Not known | Functioning PGL |
| Choat et al (2014)(38) | 11 years | c.541-2A>G | NA | One day history of periumbilical abdominal pain, non-bloody and non-bilious emesis, and decreased oral intake, on a two month background of headaches upon waking | Functioning abdominal PGL |
| Martucci et al (2015)(37) | 9 years | Exon 1 deletion | NA | Symptoms of catecholamine excess | Functioning bladder PGL, multiple other tumours |
|  | 8 years | c.418G>T | (p.Val140Phe) | Symptoms of catecholamine excess, haematuria | Functioning multiple PGL including bladder PGL with metastatic spread to bone and lymph nodes |
|  | 18 years | c.380T>G | (p.Ile127Ser) | Symptoms of catecholamine excess | Functioning bladder PGL with metastases to bone and lymph nodes |
|  | 12 years | c.418G>T | (p.Val140Phe) | Symptoms of catecholamine excess, haematuria | Functioning bladder PGL with metastases to bone and lymph nodes |
|  | 15 years | c.445–447delCAinsGGTATCT | (p.Gln149LeufsX159) | Symptoms of catecholamine excess | Functioning bladder PGL with metastases to bone |
| Cascón et al (2008)(48) | 17 years | Exon 1 deletion | NA | Not known | Abdominal PGL and renal oncocytoma, with metastases to bone 10 years later |
| Cascón et al (2013)(39) | 12 years | c.10413-73-3866del | NA | Headache, palpitations | Thoracic/abdominal PGL |
|  | 14 years | c.10413-73-3866del | NA | Not known | Two thoracic/abdominal PGLs |
|  | 17 years | c.10413-73-3866del | NA | Not known | PCC, thoracic/abdominal PGLs, HNPGL, metastases at 24 years to bone, lung and ovary |
|  | 14 years | c.10413-73-3866del | NA | Local pain, diaphoresis, headache | Thoracic/abdominal PGL |
|  | 14 years | c.10413-73-3866del | NA | Diaphoresis, headache | Functioning thoracic/abdominal PGL |
|  | 12 years | c.166-170delCCTCA | (p.Pro56delTyrfs5) | Diaphoresis, headache | PCC, two HNPGLs |
|  | 13 years | c.166-170delCCTCA | (p.Pro56delTyrfs5) | Diaphoresis, local pain, fatigue, fever, headache | Two non-functioning thoracic/abdominal PGLs, with metastases to liver |
|  | 14 years | c.166-170delCCTCA | (p.Pro56delTyrfs5) | Local pain, vertigo | Thoracic/abdominal PGL |
|  | 12 years | c.166-170delCCTCA | (p.Pro56delTyrfs5) | Not known | Thoracic/abdominal PGL |
|  | 13 years | c.540G>C | NA | Not known | Functioning PCC |
|  | 12 years | c.644delC | (p.Tyr216Ilefs4) | Dyspnoea, local pain | Thoracic/abdominal PGL |
|  | 14 years | c.49A>G | (p.Thr17Ala) | Diaphoresis, headache, palpitations, heat sensitivity | Functioning PCC |
| Lefebvre et al (2012)(40) | 12 years | NA | NA | Not known | Sporadic thoracic/abdominal/pelvic PGL |
|  | 13 years | c.166_170del | (p.Pro56TyrfsX5) | Not known | Thoracic/abdominal/pelvic PGL |
|  | 15 years | c.423+1G>C | NA | Not known | Thoracic/abdominal/pelvic PGL |
|  | 17 years | c.688C>T | (p.Arg230Cys) | Not known | Sporadic PCC |
|  | 10 years | c.713del | (p.Phe238SerfsX10) | Not known | PCC |
|  | 18 years | c.724C>T | (p.Arg242Cys) | Not known | PCC |
| Hammond et al (2010)(52) | 11 years | NA | NA | Facial palsy | PGL |
| Muth et al (2012)(42) | 15 years | c.IVS4+1G>A | NA | Not known | Functioning PGL |
| Musil et al (2010)(43) | 13 years | c.589_600dup | (p. Cys196 Cys200dup) | Diaphoresis, headaches | Functioning abdominal and mediastinal PGL |
| Timmers et al (2007)(17) | 10 years | c.73-9A>G | NA | Headache, diaphoresis, flushing | Abdominal/pelvic and neck PGL with metastases to the lung, liver and bone |
|  | 10 years | c.136C>T | (p.Arg46X) | Headache, tachycardia/palpitations, diaphoresis, anxiety/panic attacks, nausea, chest pain, fatigue | Abdominal/pelvic and mediastinal PGL with metastases to lung and bone |
|  | 11 years | c.418G>T | (p.Val140Phe) | Headache, tachycardia/palpitations, diaphoresis, flushing, pallor, anxiety/panic attacks, dizziness, nausea, tumour-related pain, exercise-related symptoms, fatigue, weakness | Abdominal/pelvic PGL with metastases to lungs and bone |
|  | 7 years | c.689G>A | (p.Arg230His) | Headache, tachycardia/palpitations, diaphoresis, pallor, tremor, anxiety/panic attacks, nausea, tumour-related pain, hypertensive crisis including stroke | Abdominal/pelvic PGL with metastases to lungs and bone |
| Mora et al (2006)(46) | 13 years | c.300_304delCCTCA | (p.Pro56delTyrfsX5) | Abdominal pain, fatigue, prolonged fever | Functioning abdominal and mediastinal PGL with metastases to hepatic nodes |
|  | 10 years | c.778delC | NA | Sudden dyspnoea | Functioning paravertebral PGL |
|  | 16 years | c.300_304delCCTCA | (p.Pro56delTyrfsX5) | Headache and vertigo during urinary voiding | Pelvic PGL |
| Majumdar et al (2010)(50) | 13 years | c.418G>T and c.200+7A>G | (p.Val140Phe) and splice site | Severe headaches on a background of delayed puberty and intermitted nocturnal enuresis | Functioning abdominal PGL with metastases to the bone |
| Armstrong et al (2009)(51) | 13 years | Complete SDHB gene deletion | NA | Dysmenorrhoea | Functioning composite PGL/neuroblastoma |
| Ghayee et al (2009)(49) | 18 years | c.725G>A | (p.Arg242His) | Palpitations, chest pain, gait abnormalities | Functioning mediastinal PGL with metastases to spine and psoas |
|  | 18 years | c.286+2T>A | NA | Not known | Functioning mediastinal PGL |
| Daniel et al (2016)(45) | 15 years | c.72+1G>T | NA | Not known | Functioning pelvic PGL |
|  | 17 years | c.600G>T | (p.Trp200Cys) | Not known | Multiple functioning pelvic PGL |
| Hermsen et al (2010)(54) | 16 years | c.312insCACTGCA | (p.Ile105HisfsX16) | Not known | Jugular PGL |
| Neychev et al (2015)(55) | 15 years | c.725G>A | (p.Arg242His) | Enlarging neck mass, dysphagia, weight loss, snoring, headaches, diaphoresis, pre-existing developmental delay | Functioning HNPGL and para-aortic PGL with metastases to bone |
| Kim et al (2009)(56) | 15 years | c.640C>T | (p.Gln214X) | Hypertensive crisis with headache, nausea, vomiting, palpitations | Functioning retroperitoneal, extra-adrenal PGL |
| Prasad et al (2009)(57) | 13 years | c.88delC | (p.Gln30Argfs) | Diaphoresis, flushing, headaches, lightheadedness, fatigue, vomiting | Multiple functioning retroperitoneal PGL, nephrogenic adenoma |
| Sait et al (2017)(58) | 13 years | c.286+2T>A | NA | Right-sided abdominal pain, intermittent constipation | Non-functioning PCC with metastases to bone |
| Cascon et al (2005)(59) | 14 years | Exon 1 deletion | NA | Not known | Functioning retroperitoneal PGL |
|  | 18 years | Exons 1-8 deletion | NA | Not known | Functioning HNPGL, renal angiolipoma |
| **SDHC Mutations** | | | | | |
| Domingues et al (2012)(41) | 17 years | c.77+36T>A | NA | Not known | Functioning, malignant abdominal PGL |
| Dimachkieh et al (2018)(53) | 8 years | c.397C>T | (p.Arg133X) | Stridor on a background of wheezing and exertional dyspnoea | Non-functioning tracheal PGL |
| Else et al (2014)(60) | 15 years | c.405+1G>C | NA | Not known | Non-functioning HNPGL |
| **SDHD Mutations** | | | | | |
| Neumann et al (2004)(8) | 5 years | c.14G>A | (p.Trp5X) | Not known | Functioning PCC |
|  | 18 years | c.14G>A | (p.Trp5X) | Not known | Non-functioning HNPGL |
|  | 13 years | c.33C>A | (p.Cys11X) | Not known | Functioning adrenal and extra-adrenal PCC |
|  | 15 years | c.204-216del13bp | NA | Not known | Functioning PCC |
|  | 15 years | c.361C>T | (p.Gln121X) | Not known | Functioning PCC |
|  | 13 years | c.441delG | NA | Not known | Functioning adrenal and extra-adrenal PCC |
| Fish et al (2007)(14) | 18 years | c.112C>T | (p.Arg38X) | Not known | Bilateral HNPGLs, and kidney, adrenal gland, retroperitoneum and paraspinal PGLs |
| Pandit et al (2016)(36) | 18 years | c.386_386delT | (p.Leu129TrpfsX6) | Not known | Functioning PCC |
| Cascón et al (2013)(39) | 14 years | c.129G>A | (p.Trp43X) | Not known | Non-functioning bilateral carotid PGL with lung metastases 10 years later |
|  | 11 years | c.302T>C | (p.Leu101Pro) | Hypoacusis | Two non-functioning HNPGLs |
|  | 17 years | c.334-337delACTG | (p.Asp113Metfs21) | Not known | Bilateral carotid PGL, PCC, thoracic/abdominal PGL |
|  | 18 years | c.64C>T | (p.Arg22X) | Not known | Non-functioning bilateral carotid PGL |
| Lefebvre et al (2012)(40) | 15 years | c.206_218del | (p.Glu69ValfsX13) | Not known | PCC and thoracic/abdominal/pelvic PGL |
|  | 17 years | c.337_340del | (p.Asp113MetfsX21) | Not known | PCC |
| Domingues et al (2012)(41) | 15 years | Exon 1 deletion | NA | Not known | Functioning abdominal PGL |
|  | 9 years | c.127G>C | (p.Ala43Pro) | Not known | Functioning abdominal PGL and PCC |
| Neumayer et al (2007)(44) | 16 years | c.271_282del | NA | Headache, vertigo, tinnitus | Functioning carotid, thoracic, adrenal and extra-adrenal PGL |
| Novosel et al (2004)(47) | 13 years | c.13838delG | NA | Abdominal pain, vomiting, weight loss | Multiple functioning PCCs and PGLs |
| Schiavi et al (2006)(61) | 15 years | c.341G>C | (p.Tyr114Cys) | Not known | Jugulotypmanic PGL, bilateral carotid body tumour |
| Marvin et al (2009)(62) | 15 years | c.94_95delTC | (p.Ala33Ilefs) | Severe depression, attention deficit hyperactivity disorder (ADHD), postural presyncope, occasional non-pulsatile tinnitus, 2 years of intermittent thick phlegm and non-seasonal rhinorrhoea | Non-functioning HNPGL |
| Srirangalingam et al (2010)(63) | 15 years | c.169+1G>A | NA | Palpitations, diaphoresis | Functioning PCC, PGL |
| Timmers et al (2008)(64) | 10 years | c.443G>A | (p.Gly148Asp) | Not known | Functioning HNPGL with metastases to bone, liver, lung and lymph nodes |
| Badenhop et al (2004)(65) | 14 years | c.276_278delCTA | (p.Tyr93del) | Not known | HNPGL |
| Fakhry et al (2008)(66) | 18 years | c.418delT | (p.Cys140AlafsX28) | Not known | Bilateral carotid body, abdominal and jugulo-tympanic PGL |
| Gimm et al (2000)(67) | 17 years | c.34G>A | (p.Gly12Ser) | Not known | Malignant functioning PCC |
